# Constructing the Energy Landscape for Genetic Switching System Driven by Intrinsic Noise

**DOI:** 10.1371/journal.pone.0088167

**Published:** 2014-02-13

**Authors:** Cheng Lv, Xiaoguang Li, Fangting Li, Tiejun Li

**Affiliations:** 1 School of Physics, Peking University, Beijing, China; 2 LMAM and School of Mathematical Sciences, Peking University, Beijing, China; 3 Center of Quantitative Biology, Peking University, Beijing, China; 4 Beijing International Center for Mathematical Research, Beijing, China; University of Adelaide, Australia

## Abstract

Genetic switching driven by noise is a fundamental cellular process in genetic regulatory networks. Quantitatively characterizing this switching and its fluctuation properties is a key problem in computational biology. With an autoregulatory dimer model as a specific example, we design a general methodology to quantitatively understand the metastability of gene regulatory system perturbed by intrinsic noise. Based on the large deviation theory, we develop new analytical techniques to describe and calculate the optimal transition paths between the on and off states. We also construct the global quasi-potential energy landscape for the dimer model. From the obtained quasi-potential, we can extract quantitative results such as the stationary distributions of mRNA, protein and dimer, the noise strength of the expression state, and the mean switching time starting from either stable state. In the final stage, we apply this procedure to a transcriptional cascades model. Our results suggest that the quasi-potential energy landscape and the proposed methodology are general to understand the metastability in other biological systems with intrinsic noise.

## Introduction

Stochasticity is an inherent property of living cells. Especially when the low copy number of species like the DNA and mRNA are taken into account, stochastic fluctuations can become significant and may qualitatively affect the behavior of the whole system [1], [2]. To deal with these fluctuations, cells have evolved many mechanisms, of which genetic switch is a typical example. Cellular systems performing genetic switches usually consists of one positive feedback or double negative feedbacks [3], [4]. Depending on the robustness of the feedbacks, cells can perform switches either spontaneously or on call [5].

Previous kinetic studies about cellular stochasticity have been formulated by using the generating function [6], system size expansion [7], [8], large deviation theory (LDT) [9]–[13], or by employing WKB approximation to the chemical master equations (CMEs) [14], [15], etc. However, only few of them take transcriptional noise into account explicitly. Some recent studies have shown that correlations between mRNA and protein levels do not always perform equally well in revealing genetic regulatory relationships [16], [17], and the involvement of mRNA has a large effect on the switching times [18], [19]. On the other hand, ever since Waddington’s “epigenetic landscape” proposed in 1957 [20], the energy landscape have been widely used to provide intuitive illustration of the dynamics and evolution of genetic regulatory systems [1], [11], [21]. Thus it is important and desired to have an approach which can effectively determine the key features of a noisy gene expression system, such as constructing the corresponding “Waddington potential”, identifying the transition paths between metastable states and computing the transition rates, etc.

In this paper, we present a methodology to understand the metastability of the genetic switches in gene expression driven by the intrinsic noise based on LDT for Markov processes [22]–[24]. By explicitly taking mRNA noise into account, we obtain the most probable transition paths for off-to-on and on-to-off genetic switches through the geometric minimum action method (gMAM) [25]. Furthermore, we construct the global quasi-potential energy landscape, which is the rationalized version of the Waddington potential in this context. Based on the obtained quasi-potential, we obtain quantitative results for transition rates between metastable states and the intrinsic noise strength of gene expression state. We also consider the reduction of redundant dimensions if we are only interested in the energy landscape for partial components of the whole system. We successfully apply this methodology to a transcriptional cascades model. The relation between our and other approaches in literature is also discussed. From the authors’ opinion, this framework is generally applicable for studying transitions between stable-saddle-stable fixed points with jump type noise generated by Gillespie type birth-death dynamics [26]. The quasi-potential energy landscape will be a powerful tool to unravel the metastable properties in more general biological processes.

## Models and Methods

**Figure 1:**
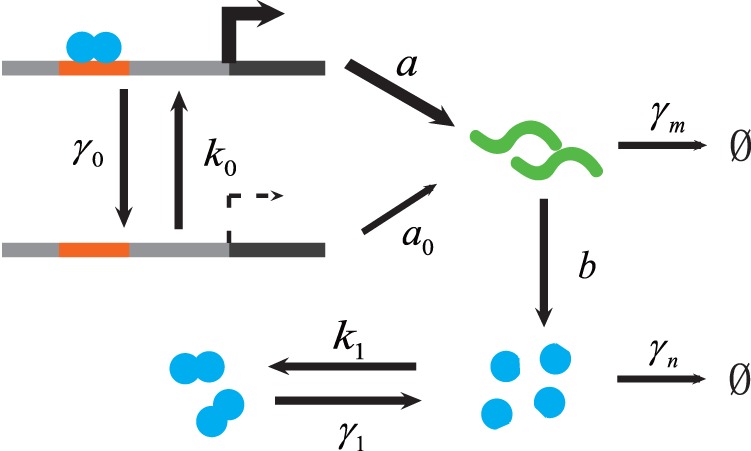
The autoregulatory dimer model with positive feedback. Promoter transitions are regulated by the dimerized transcription factor with rate 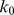 and 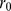. 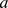 is the transcription rate of active promotor, with a very small transcription rate of inactive promotor 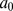. 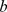 is kinetic rate of translation, 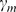 and 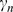 are degradation rates of mRNA and protein, 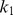 and 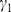 are the rates of dimerization and de-dimerization. All the processes are modeled as elementary reactions and all reaction rates are rescaled by the protein decay rate (i.e. 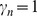 unless stated otherwise).

We illustrate our method through a specific two-state gene expression model in Fig. 1. We will refer to it as the “dimer model” throughout the remainder of this paper. In this model, the gene at the active state transcribes mRNA with a much larger rate than it at the inactive state. Proteins translated from mRNA can aggregate into dimers that bind to the promotor site of the gene via a positive feedback. All of the processes are modeled as elementary reactions and all reaction rates are rescaled by protein decay rate (i.e. we will set 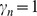 unless stated otherwise). Here we assume that the reaction rates of dimer binding and dropping from DNA are much larger than the other reactions.

### Classical Methods and Issues

The deterministic mean-field description of this dimer model through quasi-steady state approximation (QSSA) yields the ODEs.


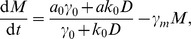



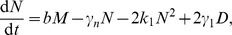



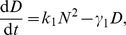


where 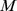, 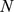 and 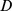 are the mean number of the mRNA, protein and dimer respectively, and the parameters are shown in Fig. 1. This system has two stable fixed points and one saddle in physically reasonable parameter regime (see Text S1:I). These two stable fixed points correspond to the expressed and unexpressed states at which the copy number of proteins is at high or low state, respectively. With this deterministic description, once the system settles in one of its two attractive fixed points, it will stay there forever. However, in the presence of intrinsic noise, the system will fluctuate around its attractive fixed points and switch between these two metastable states on a large timescale. This type of switching among metastable states under small perturbations is exactly the rare events studied in the literature. The large deviation theory (LDT) is an appropriate tool to quantitatively describe the rare transitions [23], [25], [27]. Roughly it tells that when the system size 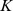 is sufficiently large, the probability that the trajectory of the stochastic dynamics 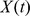 stays in a small 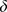-neighborhood around a specific path 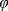 can be given as

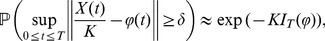
(1)where 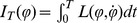 is called the rate functional. Thus the most probable transition path can be obtained by minimizing 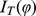 associated with the Lagrangian the 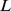. Our task is to find the 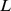 for specific models. For Gillespie type birth-death dynamics, 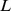 has no closed form and only its dual Hamiltonian can be obtained in the large volume limit 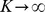, i.e. the number of all types of molecules goes to infinity. However, this approach encounters difficulty if we take the DNA switching into consideration since there is only one DNA copy in the considered model. Thus the straightforward utilization of the existed Hamiltonian in the large volume limit is invalid here.

### Large Deviation Theory

To solve this issue, we develop the LDT directly for this specific system following the way in [28] with further extension. The biologically relevant choice of parameters in our model suggests the scaling 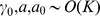, 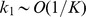 and the others are 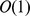, where 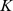 is the system size which is usually chosen as the typical number of proteins in the expressed state. This is also the correct scaling under which the mean field limit of the CMEs gives the ODE system derived from QSSA (see Text S1:I). Define the rescaled concentration variable 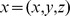 where 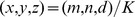 and 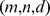 is the state vector for the number of mRNAs, proteins and dimers. Correspondingly define 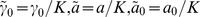 and 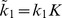 to transform all the parameters to 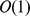 magnitude. However for notational simplicity we will drop the tilde symbol on these parameters in the rest of the paper. It turns out that the Lagrangian of our model has the form.
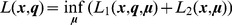
(2)which combines the LDT result for large volume limit as shown in (1) and the Donsker-Varadhan type LDT result [22], [29] for DNA fast switching. Here 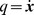 resembles the velocity in classical mechanics, 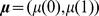 is a probabilistic 2-vector describes the residence distribution of DNA at the inactive or active state.

Let us illustrate the construction of (2) via an intuitive way as follows. The net effect of the fast switching of DNA induces a residence distribution 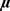 with components 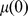 and 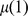 characterizing the probability of DNA staying at the inactive and active states, respectively. Whenever DNA is at the inactive or active state, we can apply the traditional LDT result in the large volume limit. This leads to the first part 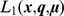 in (2). The second part describes how the visiting distribution induced by the random fast switching of DNA is close to a prescribed residence distribution 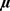 given the current state 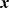. This is exactly what the Donsker-Varadhan type LDT gives [22], [29], which is described by 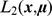. Since we are only interested in the LDT for the state variable 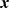, the overall Lagrangian should be taken infimum with respect to all of the possible residence distributions 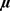. All of the statements will be made clear in the continued paragraphs.

Similar as the case in the large volume limit, it is not feasible to get the explicit form of the Lagrangian 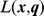 in general, but its dual Hamiltonian 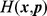 can be available, where 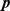 is the generalized momentum conjugate to 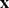 as in classical mechanics. They are connected via the Legendre transform.


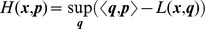



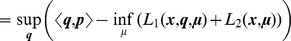



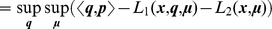



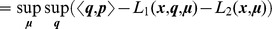




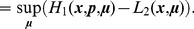
(3)


For the Gillespie type birth-death process with 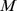 reaction channels and the propensity function 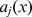 and stoichiometric vector 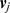 for 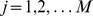, the existed LDT result gives the Hamiltonian [23].
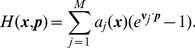
(4)


Specifically in our model we have.
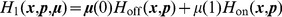
(5)with the Hamiltonian
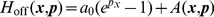
(6)when the DNA is at the inactive state and
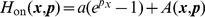
(7)when the DNA is at the active state. Here 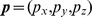 and 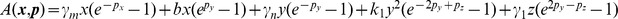 corresponds to the part of the Hamiltonian for the gene expression, i.e. the transcription and translation processes. On the other hand, the famous Donsker-Varadhan LDT gives the Lagrangian
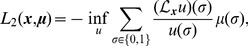
(8)where 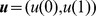 is any 2-vector and 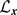 is the infinitesimal generator for the DNA two-states jumping process at a given state 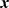 defined as



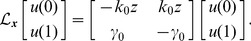
(9)The direct calculation shows that
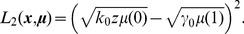
(10)


Combining Eqs. (3), (5), (6), (7) and (10), we obtain the final explicit Hamiltonian by optimization.
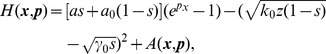
(11)where 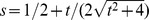 and 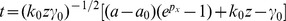. This derivation can be easily extended to similar problems.

It is worth noting that one can show the Hessian of 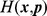 with respect to 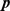 has the form.
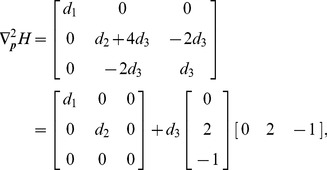
(12)where 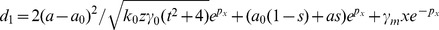, 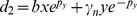 and 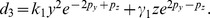 Since 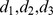 are positive when 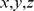 are positive, the decomposition in (12) shows that the Hessian is positive definite. This means the Hamiltonian is convex with respect to 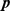 in physically meaningful domain. It is quite different from that obtained by WKB asymptotics [15] (See Text S1: II). The convexity of the Hamiltonian is testified to be essential for the robustness and efficiency of the numerical algorithm [25] both theoretically and practically. It can be rigorously proved that it is a natural by-product from the LDT analysis.

### Quasi-Potential and Optimal Transition Path

With the obtained LDT, we can get the optimal transition path through variational optimization. Moreover, we can define the local quasi-potential 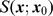 with respect to a meta-stable state 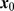 as.
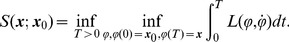
(13)


From classical mechanics, the local quasi-potential 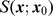 satisfies a steady-state Hamilton-Jacobi equation characterized by the Hamiltonian shown in Equation (11).
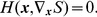
(14)


The LDT also confirms the equilibrium distribution of the system through a global quasi-potential function 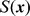.
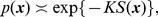
(15)where the symbol 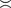 means the equality relation on a logarithmic scale, and 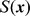 can be obtained from its local version 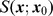 by a suitable sticking procedure which we will describe in the next subsection. This function 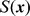, which naturally serves as a rationalized version of the Waddington potential, is one main point of this article.

The classical Hamilton-Jacobi theory enables one to solve the local quasi-potential 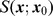 satisfying (14) with variational methods. Here we employ the powerful geometric minimum action method (gMAM) proposed in [25] to compute 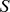 by minimizing the action functional with a prescribed Hamiltonian (11). The key idea of gMAM is essentially the Maupertuis principle in classical mechanics, which reformulates the action functional on the space of curves with intrinsic parameter, thus frees the time variable in the minimization process and still keeps its efficiency in high dimensions. This approach also resolves the issue of the singular boundary value problem by solving Hamilton’s equations directly [15], [30]. Specifically after each run of gMAM with fixed starting and ending points, one obtains the minimized action 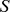 and the corresponding optimal path. The readers may be referred to Text S1:IV for more details.

It is worth asking whether the choice of the large parameter 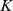 affects the final results since any choice is artificial in practice. An affirmative answer is given in Text S1:III that only the scaling matters and the final systems are equivalent with respect to different choices of the large parameter 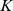.

### Construction of Global Quasi-Potential Energy Landscape

Based on the obtained local quasi-potential 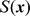 starting from the on and off states, we may construct the global quasi-potential energy landscape for genetic switching model by sticking them together. The system with only two metastable states and one saddle point, as our dimer model, can be handled conveniently as the way shown below. The readers may refer to [24] for systematic methods of sticking the global quasi-potential for more complex systems.

In our dimer model, we first compute the local quasi-potential 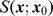 starting from two metastable states 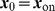 and 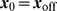. We define 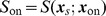 and 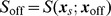, where 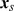 is the saddle point. Denote 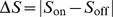. Suppose 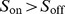, then the global quasi-potential 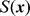 is given by.
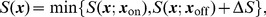
(16)otherwise 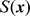 has the form



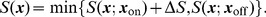
(17)It is not difficult to observe that sticking the two local quasi-potential via the linking saddle 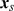 is the key point in this construction.

In most cases, the considered system is in high dimensions while we are only interested in partial variables which is in low dimensions. This is also the case in our dimer model. Although the global quasi-potential 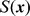 is in three dimensions, we are mainly interested in its 2D configuration in the mRNA-Protein plane. So we need to reduce the redundant dimension 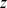 to obtain a 2-D potential 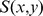. We proceed with the following arguments.

According to the LDT analysis (15), we obtain.
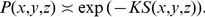
(18)With the same reason, we have
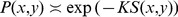
(19)where 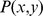 is the reduced distribution for mRNA and protein. By definition, this distribution is given by
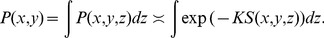
(20)From the Laplace asymptotics [29] we get a simple reduction strategy.
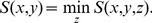
(21)


This argument is general for any high dimensional situations and indeed it is also applied to the noise cascading model considered in our later text.

## Results

### Optimal Transition Path

**Figure 2:**
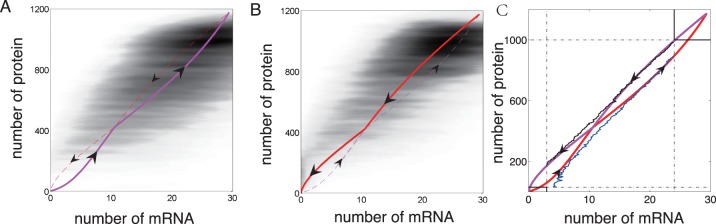
Switching paths (A) from off to on state (purple solid curve) and (B) from on to off state (red solid curve) and MC simulations for both switching trajectories. We take the two stable fixed points in the deterministic dynamics as the starting and ending points. Darkness of the shading points represents the number of visits for reactive trajectories with smoothing. (C) Averaged switching trajectories from MC simulation. For each number of protein, we average in the mRNA dimension using probability as weight. Here the statistical results around each stable state is not shown because of the restrictions by our MC simulation algorithm (see Text SI:VI-A). The results are obtained from 1000 independent long time MC simulations. The parameters here are 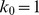, 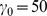, 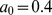, 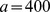, 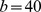, 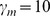, 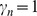, 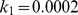, and 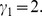

The large-deviation theory predicts that when events with little likelihood occurs, they will follow the optimal transition path which minimizes the action (13) with high probability. The probability of those paths deviated from the optimal one will decay exponentially. By choosing two stable states as the starting and ending points respectively, we obtained the switching path from either of the two states (see Fig. 2). For the convenience of visualization, we project the transition paths onto the mRNA-protein plane.

Figure 2 shows clearly that when switch occurs, the trajectory prefers to be around the most probable path characterized by the Hamiltonian (11). The fact that the off-to-on and on-to-off paths are not identical agrees with the previous studies that the switching process is irreversible. The irreversibility is fundamental in chemical reaction kinetics due to the non-gradient nature of the considered system and can be considered as a form of hysteresis. However, in contrast with the previous study [11], our results indicate that when the noise level goes to zero, both optimal transition paths pass through the same bottleneck, i.e. the saddle point obtained from the corresponding deterministic model. This suggests that the saddle point has the lowest barrier height along its stable manifold and is in accordance with the energy landscape shown later.

### Global Quasi-potential Energy Landscape

**Figure 3:**
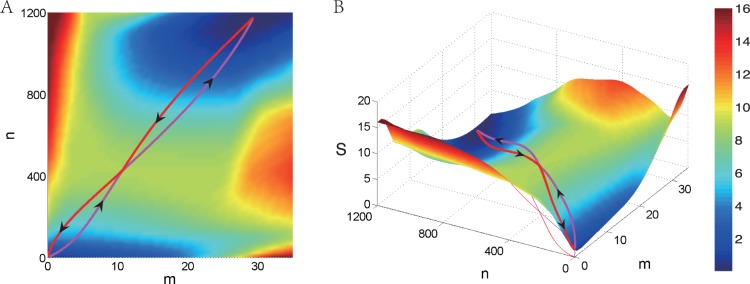
Quasipotential energy landscape of the whole genetic switching system with (A) two and (B) three dimensional view as well as switching paths between two stable fixed points. Each path passes through the saddle point. Here, the parameters are the same as in Fig. 2.

Applying the method of constructing global quasi-potential, we can compute the 2-D potential 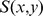 for our dimer model. The result is shown in Fig. 3.

In Fig. 3, we observe that the on and off states correspond to two local minimum on the quasi-potential energy landscape, the saddle of the deterministic dynamical system exactly corresponds to the saddle point on the quasi-potential energy landscape too. The flatness along the mRNA direction keeps in good accordance with the large fluctuation observed in the reactive trajectories.

To further characterize the switching path, we denote the first half (i.e. the part between the starting point and the saddle point) as the uphill path and the latter half as the downhill path. One may note that the transition path is also given by the Hamilton’s equations 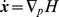, 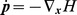. Therefore based on the fact 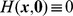, we obtained 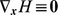 when 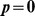. At the saddle point in any transition path, we have 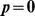 [25], and thus 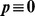 along the whole downhill path. With this result we obtain the downhill equations 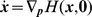, which exactly corresponds to the corresponding deterministic dynamics. This fact explains that after climbing the saddle point the biological system relaxes to its attracting state fast without costing any action. This fact was also pointed out in [30]–[32].

On the other hand, the Hamilton-Jacobi theory also yields the uphill dynamics.
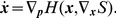
(22)


It is difficult to give a thorough understanding about the whole uphill path because of the general nonlinearity of 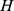. However, an analysis around the critical points is instructive. Based on the fact 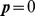 at critical points (i.e. the metastable states and saddle), we have by Taylor expansion.
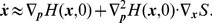
(23)


Recall that 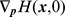 corresponds to the deterministic mean field ODEs, the equation (23) is exactly the uphill path of a chemical Langevin dynamics [33] (See more details in Text S1:VI). However, this chemical Langevin dynamics is not a straightforward generalization from the mean field ODEs like the usual large volume limit. Indeed, this reflects the specialty in our model setup which is related to the DNA fast switching. To see this more concretely, we have the approximated uphill dynamics for the 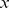-component in our dimer model as.
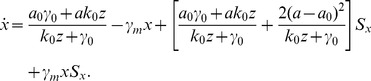
(24)


The corresponding effective Langevin dynamics for the 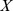-component reads.
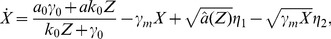
(25)where 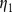 and 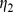 are two standard temporal Gaussian white noise with mean 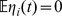 and covariance 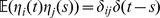, and 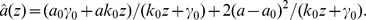 It is remarkable that besides the mean field transcription rate 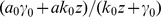, we have an additional term 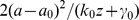 in 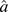. This is quite different from the usual chemical Langevin equations where for the 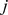-th reaction the diffusion term 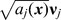 has the same form as its corresponding drift part 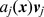 except a square root operation. The additional term in 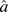 makes that the fluctuation of the transcription is larger than that in gene expression processes without DNA switching, yet has the same mean field transcription rate 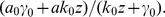 This observation coincides with the theoretical analysis in [34] although no feedback is considered there.

The quasi-potential energy landscape not only provides the pictorial illustration for the dynamical transitions, it also contains many quantitative information to understand the metastability in genetic switching models. Once the global energy landscape is obtained, one can get the stationary distribution of the whole system via Eq. (15). Furthermore, it is very easy to calculate two main characteristic quantities used to describe a genetic switching system through only small amount of computational efforts. One is the transition rates, corresponding to the lowest barrier heights between two metastable states, and the other is the noise strength, corresponding to the steepness of quasi-potential around each metastable state.

### Global Property: Mean Switching Time (MST)

The transition rate of switching systems is often characterized by Mean Switching Time (MST). We can compute the MST from either metastable state in the dimer model. For example, according to [24], the MST 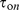 from on-to-off transition can be estimated from an asymptotic analysis.
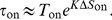
(26)


Here 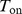 is a prefactor, and the quasi-potential energy barrier is 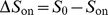, where 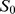 and 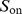 are the action values at the saddle and on states, respectively. The result for the MST of off-to-on transition 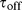 is similar. Although for one dimensional system the prefactor of MST can be obtained [31], there are no available results in high dimensions because of the geometry problem and the non-gradient nature of the system [35], [36]. Fortunately, the prefactor varies slowly in many cases, therefore we can compare the MC simulations with the exponential time part and adjust the prefactor 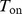 to fit the numerical results.

**Figure 4:**
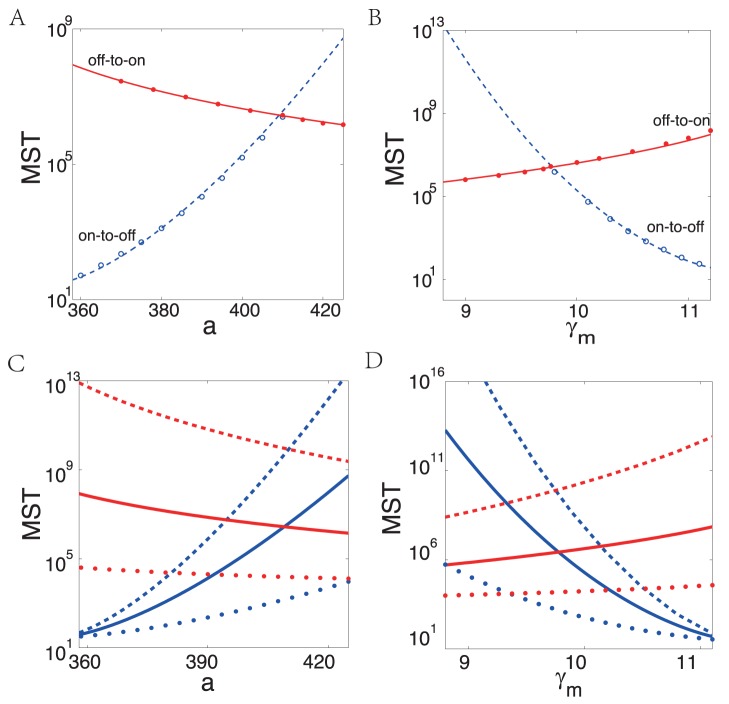
The mean switching time (MST) and quasipotential energy landscape as a function of parameters. (A) and (B): MST as a function of transcription rate 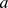. Promoter transition rates 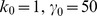, the gMAM results with numerical prefactor of off-to-on transition (red solid line) and on-to-off transition (blue dashed line), compared with MC simulations (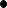) and (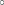), respectively. (C) and (D): The gMAM results with different promoter transition rates of off-to-on transition (red) and on-to-off transition (blue), where solid line with 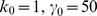 is same as (A) and (B), the faster transition rate in dashed line with 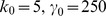, the slower transition rate in dotted line with 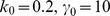. Other parameters are 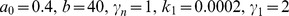; in (A,C), 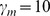 and (B,D) 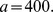

The sensitivity of both MSTs, 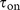 and 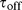, to the change of transcription rate 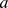 and mRNA decay rate 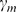 are investigated and compared with MC simulations in Fig. 4A and Fig. 4B, where the prefactors of off-to-on and on-to-off transitions are estimated as 2300 and 29, respectively. It can be observed that the MST is excellently predicted by Eq. (26) up to a slowly varying prefactor. And it is worth noting that when the MST becomes very large, the efficiency of the classical MC simulations gets extremely low while it is well kept in our approach.

The positive feedbacks in genetic circuit usually provide cellular memory or all-or-none switch. The results in Fig. 4 reveal the robustness and sensitivity of the dimer model to the change of different kinetic parameters. In Fig. 4A and Fig. 4B, the promoter transition rates 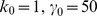. When the transcription rate 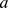 increases from 360 to 420, the MST from on-to-off states 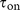 increases exponentially, while the MST of off-to-on transition 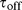 decreases slowly; it means the on-state becomes more stable while the off-state can still keep its stability. Therefore when the transcription rate 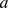 is increased, our genetic dimer circuit with positive feedback provides a stable cellular memory at the on state, but the off state remains its stability. Thus the system can not switch from the off state to the on state effectively.

**Figure 5:**
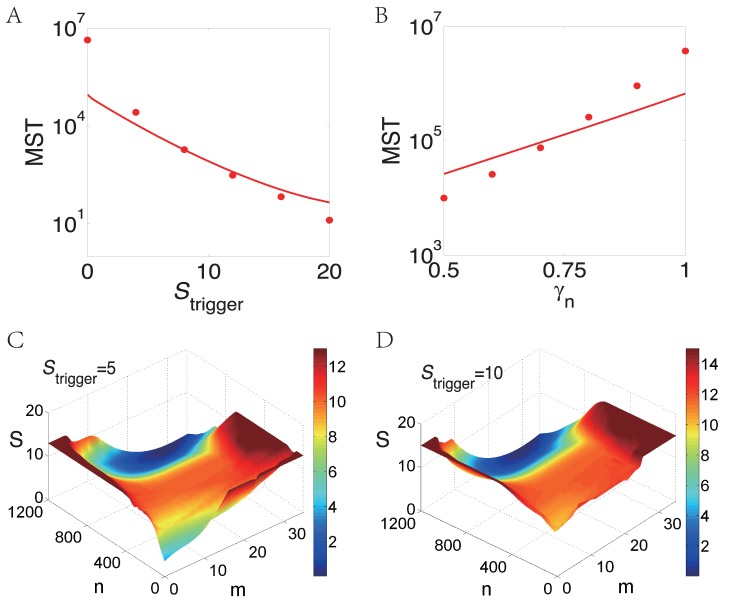
The mean switching time (MST) of off-to-on transition as a function of (A) trigger signal strength that transcribes mRNA at constant rate and (B) degradation rate of protein 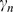. (C) and (D): Quasipotential energy landscape with different trigger strength. 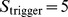 in (C), and 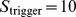 in (D). Other parameters are 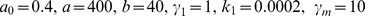; 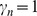 in (A,C,D), and 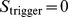 in (B).

How to turn on the genetic switch? The results in Fig. 5 provide two possible effective choices. The first choice is to pose an additional source of the mRNA production, which we call as the trigger signal. We denote the additional mRNA production rate as 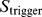. If 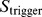 increases from 0 to 20, then MST of off-to-on transition 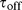 will decrease exponentially and the genetic switch is turned on. We also show how the global energy landscape changes with different trigger rate 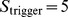 in Fig. 5C and 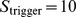 in Fig. 5D. It is evident to see from the figures that when the trigger signal increases, the barrier height from off-state to on-state decreases, which is in accordance with the MST of off-to-on transition 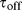. The relevant biological switch can be found in the start point of budding yeast cell cycle process, where the additional trigger signal in G1 cyclin Cln3 causes the activation of G1 transcription factor SBF and MBF [37]. The second possible choice is to decrease the degradation rate of protein 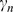. When the degradation rate of protein 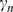 decreases from 1 to 0.5 in Fig. 5B, MST from off-to-on transition 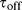 will also decrease exponentially from 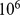 to 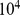 and turn on the genetic switch. This is the case in *Bacillus subtilis*, where the transitions into competent state is caused by decreasing the degradation rate of protein ComK [38]. All the other relative parameters are listed in the caption of Figures.

**Figure 6:**
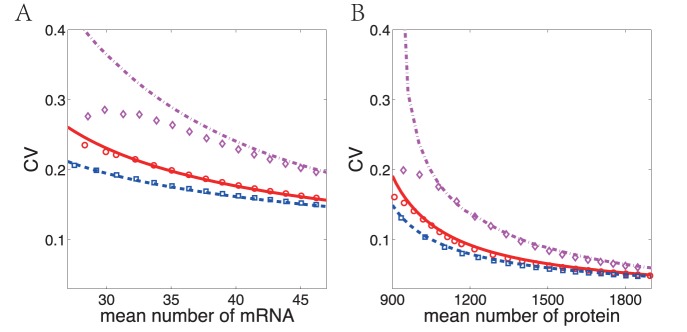
The coefficient of variation (CV) versus mean number of (A) mRNA and (B) protein induced by varying transcription rate 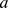 with different promoter transition rates. The lines and discrete dots correspond to analytical results and MC simulations, respectively. The results with fast promoter transition rates are shown in blue dash-dotted line and 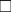, medium rates in red solid line and 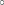, and slow rates in magenta dashed line and ◊. The parameters here are the same as in Fig. 4 (A,C).

Furthermore, we calculate the MST of both from off to on and on to off states in the different promoter transition rates. We show the results with the fast rates (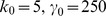) in Fig. 4C and slow rates (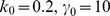) in Fig. 4D. These results indicate that the system with slow promoter transition rates tend to have short MST of off-to-on transition (red dashed lines) while fast rates lead to long MST of off-to-on transition (red dash-dotted lines). This is due to the reason that faster promoter transition rates lead to smaller mRNA and protein noise strength (see Fig. 6 for more detailed information). Ignoring the difference of the mechanism of initial transcription between prokaryotes and eukaryotes, in the simple case, the faster promoter transition rates correspond to the gene expression process in prokaryotes, and the slower promoter transition rates correspond to the slow chromatin remodeling process in eukaryotic case [39]. The results suggest that prokaryotes may have stronger cellular memory than eukaryotes.

### Local Property: Fluctuation Around Stable States

Another quantitative information that quasi-potential energy landscape can provide is the noise strength of stable states. Here we use the coefficient of variation (CV, i.e. the standard deviation over the mean) to measure the strength of fluctuation instead of the Fano factor, for the system here has positive feedback thus deviates far from Poisson statistics. Notice that the stationary distribution 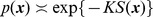, we can expand 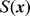 in the vicinity of high stable state 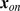 up to second order thus get the Gaussian approximation.
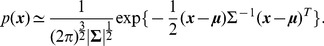
(27)


Here, 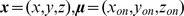, 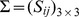, and 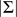 is the determinant of matrix 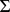. Eq. (27) holds only in the vicinity of the on state with standard deviations 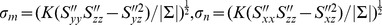 and 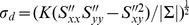. With the 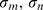 and 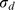 above, we can easily obtain the CV as shown in Fig. 6.

Figure 6 demonstrates that when the average expression levels increase, the noise strength of mRNA and protein decreases in our positive feedback model. The fluctuation of mRNA is usually larger than that of protein. Furthermore, the noise level with slow promoter transition rates is almost always larger than the one with fast promoter transition rates. This is in accordance with the results of MST that the system with long MST has small noise and vise versa. The inconsistent portion between analytical and simulation results (the left part of the line with slow promoter transition rates in Fig. 6B) is due to the inapplicability of Eq. (27) during the low barrier crossing process for the on state. More details may be referred to Text S1:V.

### Application in Transcriptional Cascades

To further illustrate the power of quasi-potential energy landscape and the abundant quantitative information it contains, we apply our methodology to a transcriptional cascades model based on the previous work of S. Hooshangi et al. [40]. In their work, S. Hooshangi et al. synthesized transcriptional cascades comprised of one, two, and three repression layers and analyzed the sensitivity and noise propagation as a function of network complexity. They used different concentrations of anhydrotetracycline (aTc) as inducer and measured the fluorescence intensities of protein *eyfp* (the last layer of each cascade) by the flow cytometer.

**Figure 7:**
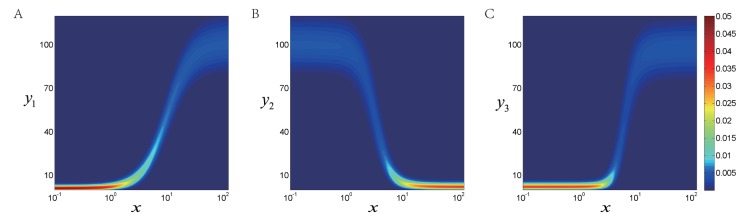
The dose response curves and probability distribution of the output protein in the 3-layer cascades (denoted by 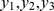) as a function of inducing signal 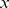, 1-layer in (A), 2-layer (B) and 3-layer (C). The probability distribution can be directly obtained from Eq. (15) after normalization. The Hill coefficient for each cascade is fitted as 2.00, 3.15 and 4.08 respectively.

Here we simplify the 3-layer cascades as 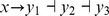, where 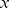 denotes the concentration of aTc as inducing signal and 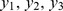 denote the output of proteins in different layers respectively. Then we directly construct the quasi-potential energy landscape for each layer and obtained the normalized probability distribution of the output to certain signal 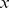 from Eq. (15). The dose response curves to increasing signal 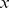 are shown in Fig. 7, which are consistent well with the previous experimental results. Further more, two features of transcriptional cascades can be observed. Firstly, the more layers the transcriptional cascades have, the sharper the response curves are (as the Hill coefficient of the 3-layer cascades is 2.00, 3.15 and 4.08 respectively). Thus the sensitivity is increased in the cascades. Secondly, the fluctuation of output can be described by the spreading width of its distribution, so more layers of cascades amplify the cell-cell variability (see Fig. S2). In short, when a cascade has more layers, its response curve gets steeper with a wider probability distribution and thus larger fluctuations. The straightforward calculation of CV based on Fig. 7 has been done and it agrees well with the MC simulations (see Text S1:VI and Figure S2).

### Limitations of The Study, Open Questions, and Future Works

We have already illustrated a general methodology based on LDT to quantitatively understand the metastability in gene expression processes perturbed by the intrinsic noise and applied it to a dimer auto-regulatory circuit model. It is clear that this methodology can be extended to more general systems, provide one can explicitly write down the Hamiltonian of the system. If all of the considered species have relatively large numbers, the Hamiltonian is simply the Eq. (4). For the case where the large volume limit fails to be true, our method is also applicable under an additional assumption that the low copy number of species reach their stationary distribution much faster than the others. This is the situation that we treat DNA in our dimer model. However, we would like to mention the limitations of our work, which of course motivates us for future studies.

The main limitations or the corresponding open questions can be summarized into the following three aspects:

1. The case where the large volume limit and the fast switching mechanism are both invalid. This prevents us to construct the LDT for the considered system. Thus there is no Hamiltonian and the current methodology fails. How to quantitatively study such systems and define the proper Waddington energy landscape is an issue.
2. The curse of dimensionality. When the problem is extended to high dimensions, although the computation of optimal transition path and the analysis of MST and CV can be achieved with a reasonable cost, constructing the whole global energy landscape is not feasible in general. However as we have argued before, the whole energy landscape is even not necessary since we are only interested in its configuration for partial components. How to extract these information directly from the Hamiltonian by smart utilization of Eq. (21) is what we are trying to do.
3. Transition rate formula in the high dimensional case. Despite the transition rate formula, i.e. the Arrhenius type formula, for the equilibrium models are well developed [41], there is no complete answer for the non-equilibrium case. The rate with form 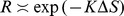 has long been established in [24], but the prefactor is not known. In one dimensional case, partial result is given [31]. But its high dimensional form is still an open question.

To understand the transition behavior for more general biological systems driven by noise, the above open problems should be overcome in the future studies.

## Conclusion and Discussion

In this paper, we have presented a methodology to construct the quasi-potential energy landscape of genetic switching system while explicitly taking mRNA noise into account. This global potential, which is a rationalized version of Waddington potential, can provide a quantitative tool to understand the metastability in more general biological processes with intrinsic noise. The results also provide some insights in gene-expression switching circuit with positive feedback, especially the robustness and sensitivity of the genetic switching system under different promotor transition rates.

For the connection with previous general methodology in literature [11], we focus more on the energy landscape and metastability properties for systems with *intrinsic noise*. Although one can principally compute the stationary distribution by solving a steady state chemical master equation on a truncated domain, our approach sufficiently utilizes the special structure of the system. Indeed, the global quasi-potential 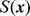 employed in this paper connects with the potential defined in [11] through 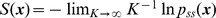, which is independent of 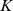. The reason we can do this is simply because the system size 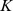 is large enough here.

With regard to the WKB and reduction approach in [15], we obtain a convex Hamiltonian based on rigorous mathematical analysis and explicitly take mRNA noise into account through the gMAM method. The convexity proves to be essential for the computational efficiency and robustness. Our derivations can be also easily extended to similar problems.

Overall, the quasi-potential energy landscape and the proposed methodology can serve as a useful tool to explore the gene expression process with intrinsic noise. Further developments such as high dimensionality issue and its applications to other biological systems like complex cellular decision making and the developmental process of cells are deserved to be investigated. The biological meaning of optimal transition path and transition states remains to be uncovered in the future studies.

## Supporting Information

Figure S1 **The network design of three synthetic transcriptional cascades.**
(EPS)

Figure S2 **Coefficient of variation as a function of mean.**
(EPS)

Text S1 **This file contains details that needed to understand the main body.** It is arranged as follows: I. Mean field limit of CMEs, II. Comparison of Hamiltonian, III. Scale independence on the choice of system size, IV. Introduction of the gMAM, V.Stochastic Simulation, VI. Analysis of Uphill Path, VII. Application in Transcriptional Cascades.
(PDF)
