# Supplementary material for: Constructing the Energy Landscape for Genetic Switching System Driven by Intrinsic Noise
Source: PLoS One. 2014 Feb 13;9(2):e88167. doi: 10.1371/journal.pone.0088167 (PMC3923795; doi:10.1371/journal.pone.0088167)
Supplement: Text S1 — This file contains details that needed to understand the main body. It is arranged as follows: I. Mean field limit of CMEs, II. Comparison of Hamiltonian, III. Scale independence on the choice of system size, IV. Introduction of the gMAM, V.Stochastic Simulation, VI. Analysis of Uphill Path, VII. Application in Transcriptional Cascades. (PDF) [file pone.0088167.s003.pdf]

Text S1
Constructing The Energy Landscape for Genetic Switching System Driven by Intrinsic Noise: Supporting Information

Cheng Lv, Xiaoguang Li, Fangting Li, and Tiejun Li

Content

I. Mean field limit of CMEs
II. Comparison of Hamiltonian
III. Scale independence on the choice of system size
IV. Introduction of the gMAM
IV-A. The Outer Loop
IV-B. Evaluating the Action
IV-C. The Inner Loop (Computing $\hat{\vartheta}(\varphi,\varphi')$)
V.Stochastic Simulation
V-A. Transition Trajectory from MC Simulation
V-B. Mean Switching Time (MST) from MC simulation
V-C. Local Property: Fluctuation around Stable States
VI. Analysis of Uphill Path
VII. Application in Transcriptional Cascades

I. MEAN FIELD LIMIT OF THE CME

In this section we take the singular perturbation analysis [1] to analyze the large system size limit of the stochastic equations when K goes to infinity. This result gives the rationale in what sense that the deterministic dynamics is related to the stochastic dynamics.

At first the backward operator $\mathcal{L}$, i.e. the infinitesimal generator of the considered chemical reaction process (also the adjoint operator arising in chemical master equations (CMEs)) has the form

$$\begin{aligned}\mathcal{L}h(0,M,N,D) =& k_0 D\,[h(1,M,N,D)-h(0,M,N,D)] + \mathbf{A}h(0,M,N,D) + a_0(\mathbf{E}_M^1-1)h(0,M,N,D),\\ \mathcal{L}h(1,M,N,D) =& \gamma_0\,[h(0,M,N,D)-h(1,M,N,D)] + \mathbf{A}h(1,M,N,D) + a(\mathbf{E}_M^1-1)h(1,M,N,D),\end{aligned} \tag{1}$$

where h is any function of 4-tuple $(\alpha,M,N,D)\in\{0,1\}\times\mathbb{N}^3$. M,N,D represent the number of mRNAs, proteins, and dimers respectively. $\alpha=0,1$ represent the inactive and active state of DNA, respectively. $\mathbf{E}_M^i,\mathbf{E}_N^j,\mathbf{E}_D^k$ are corresponding raising operators that $\mathbf{E}_M^i f(M,N,D)=f(M+i,N,D)$. The operator $\mathbf{A}$ is defined as

$$\begin{aligned}\mathbf{A}f(M,N,D) =& \gamma_N N(\mathbf{E}_N^{-1}-1)f(M,N,D) + \gamma_m M(\mathbf{E}_M^{-1}-1)f(M,N,D) + bM(\mathbf{E}_N^1-1)f(M,N,D)\\ &+k_1 N(N-1)(\mathbf{E}_N^{-2}\mathbf{E}_D^1-1)f(M,N,D) + \gamma_1 D(\mathbf{E}_N^2\mathbf{E}_D^{-1}-1)f(M,N,D).\end{aligned} \tag{2}$$

Taking the adjoint of $\mathcal{L}$, we get the CMEs for this system. Denote P_{mnd},Q_{mnd} the probability distribution function (PDF) of inactive/active DNA state with m mRNAs, n proteins and d dimers at time t. The CMEs have the form:

$$\begin{cases}\dot{P}_{mnd} = \mathcal{L}^*P_{mnd} = \gamma_0\mathbf{E}_d^{-1}Q_{mnd} - k_0 dP_{mnd} + [\mathbf{A}^* + a_0(\mathbf{E}_m^{-1}-1)]P_{mnd},\\ \dot{Q}_{mnd} = \mathcal{L}^*Q_{mnd} = -\gamma_0 Q_{mnd} + k_0\mathbf{E}_d^1(dP_{mnd}) + [\mathbf{A}^* + a(\mathbf{E}_m^{-1}-1)]Q_{mnd},\end{cases} \tag{3}$$

where $\mathbf{A}^*$ is the adjoint of $\mathbf{A}$. That is,

$$\mathbf{A}^* = \gamma_m(\mathbf{E}_m^1-1)m + bm(\mathbf{E}_n^{-1}-1) + \gamma_n(\mathbf{E}_n^1-1)n + k_1(\mathbf{E}_n^2\mathbf{E}_d^{-1}-1)n(n-1) + \gamma_1(\mathbf{E}_n^{-2}\mathbf{E}_d^1-1)d.$$

To investigate the evolution of the concentration variables, we consider the variables

$$x = M/K,\quad y = N/K,\quad z = D/K \tag{4}$$

on the rescaled lattice $\mathbb{N}^3/K$. Correspondingly, the infinitesimal generator $\tilde{\mathcal{L}}$ on the rescaled lattice has the form

$$\begin{aligned}\tilde{\mathcal{L}}h(0,x,y,z) =& Kk_0 z\left[h(1,x,y,z)-h(0,x,y,z)\right] + K\tilde{\mathbf{A}}h(0,x,y,z) + K\tilde{a}_0(\mathbf{E}_M^1-1)h(0,x,y,z),\\ \tilde{\mathcal{L}}h(1,x,y,z) =& K\tilde{\gamma}_0\left[h(0,x,y,z)-h(1,x,y,z)\right] + K\tilde{\mathbf{A}}h(1,x,y,z) + K\tilde{a}(\mathbf{E}_M^1-1)h(1,x,y,z),\end{aligned} \tag{5}$$

where the rescaled operator

$$\begin{aligned}\tilde{\mathbf{A}}f(x,y,z) =& \gamma_n y(\mathbf{E}_N^{-1}-1)f(x,y,z) + \gamma_m x(\mathbf{E}_M^{-1}-1)f(x,y,z) + bx(\mathbf{E}_N^1-1)f(x,y,z)\\ &+\tilde{k}_1 y^2(\mathbf{E}_M^{-2}\mathbf{E}_D^1-1)f(x,y,z) + \gamma_1 z(\mathbf{E}_N^2\mathbf{E}_D^{-1}-1)f(x,y,z).\end{aligned} \tag{6}$$

Define $u(x,y,z) = h(0,x,y,z), v(x,y,z) = h(1,x,y,z), \varepsilon = K^{-1}$, then we have the backward equations for the rescaled dynamics through Taylor expansion

$$\begin{aligned}\frac{du}{dt} =& \varepsilon^{-1}k_0 z(v(x,y,z)-u(x,y,z)) + \mathcal{A}u + \tilde{a}_0\partial_x u + o(\varepsilon),\\ \frac{dv}{dt} =& \varepsilon^{-1}\tilde{\gamma}_0(u(x,y,z)-v(x,y,z)) + \mathcal{A}v + \tilde{a}\partial_x v + o(\varepsilon),\end{aligned} \tag{7}$$

where

$$\tilde{\gamma}_0 = \gamma_0/K, \tilde{a} = a/K, \tilde{a}_0 = a_0/K, \tilde{k}_1 = Kk_1. \tag{8}$$

The operator $\mathcal{A}$ is the differentiation form of $\tilde{\mathbf{A}}$ as

$$\begin{aligned}\mathcal{A}f(x,y,z) =& -\gamma_n y\partial_y f(x,y,z) - \gamma_m x\partial_x f(x,y,z) + bx\partial_y f(x,y,z)\\ &+\tilde{k}_1 y^2(-2\partial_y f(x,y,z) + \partial_z f(x,y,z)) + \gamma_1 z(2\partial_y f(x,y,z) - \partial_z f(x,y,z)).\end{aligned} \tag{9}$$

From (7), the slow scale equation can be obtained by a weighted summation

$$\begin{aligned}\frac{d[\tilde{\gamma}_0 u + k_0 z v]}{dt} =& (bx - \gamma_n y - 2\tilde{k}_1 y^2 + 2\gamma_1 z)(k_0 z\partial_y v + \tilde{\gamma}_0\partial_y u) - \gamma_m x(k_0 z\partial_x v + \tilde{\gamma}_0\partial_x u)\\ &+\tilde{a}_0\tilde{\gamma}_0\partial_x u + \tilde{a}k_0 z\partial_x v + (\tilde{k}_1 y^2 - \gamma_1 z)(k_0 z\partial_z v + \tilde{\gamma}_0\partial_z u) + o(\varepsilon).\end{aligned} \tag{10}$$

By a perturbation expansion

$$\begin{aligned}u(x,y,z) &= u_0(x,y,z) + \varepsilon u_1(x,y,z) + \varepsilon^2 u_2(x,y,z) + o(\varepsilon^2),\\ v(x,y,z) &= v_0(x,y,z) + \varepsilon v_1(x,y,z) + \varepsilon^2 v_2(x,y,z) + o(\varepsilon^2),\end{aligned}$$

we find that the leading term $O(\varepsilon^{-1})$ of (7) gives

$$u_0(x,y,z) = v_0(x,y,z) \tag{11}$$

and (10) gives

$$\frac{du_0(x,y,z)}{dt} = (bx - \gamma_n y - 2\tilde{k}_1 y^2 + 2\gamma_1 z)\partial_y u_0 - \gamma_m x\partial_x u_0 + \frac{\tilde{a}_0\tilde{\gamma}_0 + \tilde{a}k_0 z}{\tilde{\gamma}_0 + k_0 z}\partial_x u_0 + (\tilde{k}_1 y^2 - \gamma_1 z)\partial_z u_0. \tag{12}$$

This equation exactly corresponds to the backward equation of the deterministic process

$$\begin{aligned}\frac{dx}{dt} =& \frac{\tilde{a}_0\tilde{\gamma}_0 + \tilde{a}k_0 z}{\tilde{\gamma}_0 + k_0 z} - \gamma x,\\ \frac{dy}{dt} =& bx - \gamma_n y - 2\tilde{k}_1 y^2 + 2\gamma_1 z,\\ \frac{dz}{dt} =& \tilde{k}_1 y^2 - \gamma_1 z.\end{aligned} \tag{13}$$

Transforming back to M, N, D variables according to (4), we obtain

$$\begin{aligned}\frac{dM}{dt} =& \frac{a_0\gamma_0 + ak_0 D}{\gamma_0 + k_0 D} - \gamma_m M,\\ \frac{dN}{dt} =& bM - \gamma_n N - 2k_1 N^2 + 2\gamma_1 D,\\ \frac{dD}{dt} =& k_1 N^2 - \gamma_1 D.\end{aligned} \tag{14}$$

This is exactly the mean field ODEs satisfied by the number of mRNAs, proteins and dimers.

The steady states of Eq. (14) satisfy

$$\begin{cases} \dfrac{\gamma_m k_0 k_1 b^2}{\gamma_1 \gamma_n^2} M^3 - \dfrac{a k_0 k_1 b^2}{\gamma_1 \gamma_n^2} M^2 + \gamma_0 \gamma_m M - a_0 \gamma_0 = 0, \\ N = \dfrac{b}{\gamma_n} M, \\ D = \dfrac{k_1}{\gamma_1} M^2. \end{cases} \tag{15}$$

It is easy to find that the system (15) has three positive solutions. We denote them as (M_i, N_i, D_i) $(i = 1, 2, 3)$, where $M_1 < M_2 < M_3$. A linearized stability analysis tells us these three states have different stability property. The Jacobian of ODEs (14) is

$$\begin{pmatrix} -\gamma_m & 0 & \frac{(a-a_0)k_0\gamma_0}{(\gamma_0+k_0 D)^2} \\ b & -(4k_1 N + \gamma_n) & 2\gamma_1 \\ 0 & 2k_1 N & -\gamma_1 \end{pmatrix}. \tag{16}$$

When the parameters assume values $k_0 = 1,\ r_0 = 50, a_0 = 0.4,\ a = 400,\ b = 40,\ \gamma_m = 10,\ \gamma_n = 1,\ k_1 = 0.0002$, and $\gamma_1 = 2$, the Jacobian matrix (16) has eigenvalues

$$\begin{aligned} \lambda &= -9.8224, -0.2467, -3.8710 \qquad \text{at } (M_1, N_1, D_1), \\ \lambda &= -9.9971, -0.9765, -2.0276 \qquad \text{at } (M_3, N_3, D_3). \end{aligned}$$

They are stable nodes. On the other hand, the eigenvalues

$$\lambda = -9.5176, 0.2414, -4.0625 \qquad \text{at } (M_2, N_2, D_2).$$

Thus it is a saddle.

The parameter values we used above are based on the references [2] and [3]. Their biological relevance can be referred to the Table I below (we assume the volume of a prokaryotic cell to be 3 μm^3 and the number of amino acids of one protein to be 500).

TABLE I: The biological relevance of the parameter values

Parameters	In Refs.	Unit	Transformed to our model	Normalized by γ_n	Value we used
k_0 (DNA activate)	2.8e+7 [4]	$s^{-1}M^{-1}$	0.0066	16.5	1
γ_0 (DNA inactivate)	0.022 [4]	s^{-1}	0.022	55	50
γ_m (mRNA decay)	5∼10 [5]	min (half-life)	0.0048∼0.0024	12∼6	10
γ_n (protein decay)	60 [6]	min (half-life)	0.0004	1	1
a (transcription)	45 [7]	bases/sec	0.03	75	400
b (translation)	15 [7]	residues/sec	0.03	75	40

In addition, we choose the values of parameters k_1 and γ_1 based on the Ref. [8] (the number of dimers per exponentially growing cell is around 113).

II. COMPARISON OF HAMILTONIANS

In this section, we will compare the differences between the Hamiltonian obtained by our approach and the Hamiltonian derived from WKB asymptotics, e.g. what was taken in [2].

Recall that our Hamiltonian derived from the large deviation theory [9,10] has the form

$$H(\boldsymbol{x}, \boldsymbol{p}) = A(\boldsymbol{x}, \boldsymbol{p}) + [as + a_0(1-s)](e^{p_x} - 1) - (\sqrt{k_0 z(1-s)} - \sqrt{\gamma_0 s})^2, \tag{17}$$

where $\boldsymbol{x} = (x, y, z)$, $\boldsymbol{p} = (p_x, p_y, p_z)$, $s = 1/2 + t/(2\sqrt{t^2+4})$, $t = (k_0 z \gamma_0)^{-1/2}[(a - a_0)(e^{p_x} - 1) + k_0 z - \gamma_0]$, and

$$A(\boldsymbol{x}, \boldsymbol{p}) = \gamma_m x(e^{-p_x} - 1) + bx(e^{p_y} - 1) + \gamma_n y(e^{-p_y} - 1) + k_1 y^2(e^{-2p_y + p_z} - 1) + \gamma_1 z(e^{2p_y - p_z} - 1).$$

To obtain the Hamiltonian from WKB approach, we utilize the chemical master equation (3). In steady state, we have

$$Q_{mnd} = \gamma_0^{-1}\mathbf{E}_d^1\{k_0 d - \mathbf{A}^* - a_0(\mathbf{E}_m^{-1} - 1)\}P_{mnd}. \tag{18}$$

So we get

$$(\mathbf{A}^* + a_0(\mathbf{E}_m^{-1} - 1))P_{mnd} + \gamma_0^{-1}[\mathbf{A}^* + a(\mathbf{E}_m^{-1} - 1)][k_0 d - \mathbf{A}^* - a_0(\mathbf{E}_m^{-1} - 1)]P_{mnd} = 0. \tag{19}$$

Denote $(x, y, z) = (m/K, n/K, d/K)$ and $P(x, y, z) \equiv P_{mnd}$, we obtain the continuous approximation of the CMEs by applying the same scaling in Section I

$$(\mathcal{A}^* - a_0\partial_x)P(x, y, z) + [\mathcal{A}^* - a\partial_x][k_0 z - \mathcal{A}^* + a_0\partial_x]P(x, y, z) = 0, \tag{20}$$

where $\mathcal{A}^*$ is the adjoint of $\mathcal{A}$ appeared in Section I. Plugging the WKB ansatz $P(x, y, z) \sim e^{-KS(x,y,z)}$ into (20) and keeping the leading terms, we get a Hamilton-Jacobi equation for $S(x, y, z)$ as $H^{\mathrm{WKB}}(\boldsymbol{x}, \nabla_{\boldsymbol{x}} S) = 0$ with Hamiltonian

$$H^{\mathrm{WKB}}(\boldsymbol{x}, \boldsymbol{p}) = A(\boldsymbol{x}, \boldsymbol{p}) - a_0(e^{p_x} - 1) + \gamma_0^{-1}[A(\boldsymbol{x}, \boldsymbol{p}) + a(e^{p_x} - 1)][k_0 z + a_0(e^{p_x} - 1) - A(\boldsymbol{x}, \boldsymbol{p})]. \tag{21}$$

Here $A(\boldsymbol{x}, \boldsymbol{p})$ is the same as that containted in (17). One may find at the first sight that the Hamiltonian H^{WKB} is quite different from the H in (17). Furthermore H^{WKB} is complex to be used.

We have proved in the main text that our Hamiltonian H is strictly convex and pointed out that H^{WKB} does not guarantee such a property. Indeed, in our parameter setup, we may easily find for example if $(x, y, z) = (27, 755, 5)$ and $(p_x, p_y, p_z) = (0.01, 0.01, 0.01)$, the eigenvalues of $\nabla^2_{\boldsymbol{p}} H^{\mathrm{WKB}}$ are (-653.5176,1645.6911,2143.6604). The negative eigenvalue means H^{WKB} is not convex even when x, y, z are all in the physically meaningful domain. Of course, this choice of $\boldsymbol{x}, \boldsymbol{p}$ is based on a random sampling in the phase space. One can find the area for H^{WKB} to be non-convex is much larger than one single point. It is also found in our computations that this non-convexity leads to breakdown or very slow convergence of the algorithm.

III. SCALE INDEPENDENCE ON THE CHOICE OF SYSTEM SIZE

We will prove the scale independence on the choice of system size for general chemical kinetic systems in this section. The specification to our concrete model is straightforward. The overall analysis tells us that the different choices of system size V (denoted as K in the main body of the paper) lead to equivalent results with the correct parameter rescaling, which is essential for the analysis.

Let us consider the classical set-up for the chemical reactions with infinitesimal generator

$$\mathcal{L}f(\boldsymbol{x}) = \sum_{j=1}^{M} a_j(\boldsymbol{x}; \theta)\left(f(\boldsymbol{x} + \boldsymbol{\nu}_j) - f(\boldsymbol{x})\right), \quad \boldsymbol{x} \in \mathbb{N}^N \tag{22}$$

where θ is the parameters appearing in the system. Suppose the propensity functions satisfy the scaling

$$a_j(V\boldsymbol{x}; V^\alpha\theta) = V a_j(\boldsymbol{x}; \theta) \tag{23}$$

where $\alpha \in \mathbb{R}$ corresponds to the scaling relation of the parameters with the system size V. In realistic situations, α usually takes values in $\{1, 0, -1, \ldots\}$.

For a fixed system and parameter θ_0 with different choices of system size V_i $(i = 1, 2)$, we define

$$\theta_0^{V_i} = V_i^{-\alpha}\theta_0, \quad \boldsymbol{y}^{V_i} = V_i^{-1}\boldsymbol{x}. \tag{24}$$

where the superscripts characterize the dependence on V_i instead of the power of an exponent. It is not difficult to show that the large volume limit of this process when $V_i \to \infty$ is

$$\frac{d\boldsymbol{y}^{V_i}}{dt} = \sum_{j=1}^{M} a_j(\boldsymbol{y}^{V_i}; \theta_0^{V_i})\boldsymbol{\nu}_j, \quad \boldsymbol{y}^{V_i} \in \mathbb{R}^N \tag{25}$$

The fixed points of the above reaction rate equations satisfies

$$\sum_{j=1}^{M} a_j(\boldsymbol{y}^{V_i}; \theta_0^{V_i})\boldsymbol{\nu}_j = 0 \tag{26}$$

It is straightforward to see that the fixed points $\{\boldsymbol{y}_{*,k}^{V_i}\}_{k=1}^{K}$ for two choices of the system size have the relation

$$V_1\boldsymbol{y}_{*,k}^{V_1} = V_2\boldsymbol{y}_{*,k}^{V_2}, \quad k = 1, 2, \ldots, K \tag{27}$$

from (23). The Hamiltonian in the large deviation theory has the form

$$H(\boldsymbol{y}^{V_i}, \boldsymbol{p}^{V_i}) = \sum_{j=1}^{M} a_j(\boldsymbol{y}^{V_i}; \theta_0^{V_i})\Big[\exp\big(\boldsymbol{p}^{V_i}\cdot\boldsymbol{\nu}_j\big) - 1\Big]. \tag{28}$$

The transition path connecting different fixed points in time T satisfies the Hamiltonian dynamics

$$\frac{d\boldsymbol{y}^{V_i}}{dt} = \sum_{j=1}^{M} a_j(\boldsymbol{y}^{V_i}; \theta_0^{V_i})\boldsymbol{\nu}_j \exp\big(\boldsymbol{p}^{V_i}\cdot\boldsymbol{\nu}_j\big) \tag{29}$$

$$\frac{d\boldsymbol{p}^{V_i}}{dt} = -\sum_{j=1}^{M} \nabla_{\boldsymbol{y}} a_j(\boldsymbol{y}^{V_i}; \theta_0^{V_i})\Big[\exp\big(\boldsymbol{p}^{V_i}\cdot\boldsymbol{\nu}_j\big) - 1\Big] \tag{30}$$

with boundary conditions

$$\boldsymbol{y}^{V_i}(0) = \boldsymbol{y}_{*,k_1}^{V_i}, \quad \boldsymbol{y}^{V_i}(T) = \boldsymbol{y}_{*,k_2}^{V_i}. \tag{31}$$

It is easy to observe that if

$$\Big\{\boldsymbol{y}^{V_i}(t), \boldsymbol{p}^{V_i}(t); \boldsymbol{y}_{*,k_1}^{V_i}, \boldsymbol{y}_{*,k_2}^{V_i}, a_j(\boldsymbol{y}^{V_i}; \theta_0^{V_i})\Big\} \tag{32}$$

is a solution, where the last three variables in the parenthesis represents the related boundary points and reaction rates. Then

$$\Big\{V_i\boldsymbol{y}^{V_i}(t), \boldsymbol{p}^{V_i}(t); V_i\boldsymbol{y}_{*,k_1}^{V_i}, V_i\boldsymbol{y}_{*,k_2}^{V_i}, a_j(V_i\boldsymbol{y}^{V_i}; V_i^{\alpha}\theta_0^{V_i})\Big\} \tag{33}$$

is also a solution by the scaling (23) and

$$\nabla_{\boldsymbol{y}} a_j(V\boldsymbol{y}; V^{\alpha}\theta) = \nabla_{\boldsymbol{y}} a_j(\boldsymbol{y}; \theta) \tag{34}$$

by direct differentiation with respect to $\boldsymbol{y}$ to both sides of (23). This explains the scale independence of the system with respect to the choice of system size V. The key point is that the choice of V does not matter but the scaling relation (23) is crucial for the considered system.

The above analysis can be generalized to the system with multiple parameters and condition

$$a_j(V\boldsymbol{x}; V^{\alpha_1}\theta_1, V^{\alpha_2}\theta_2, \ldots, V^{\alpha_m}\theta_m) = Va_j(\boldsymbol{x}; \theta_1, \theta_2, \ldots, \theta_m) \tag{35}$$

or more generally

$$a_j(\boldsymbol{x}; \theta_1, \theta_2, \ldots, \theta_m) = \tilde{a}_j(\boldsymbol{x}; \theta_1, \theta_2, \ldots, \theta_m) + V^{-l}b_j(\boldsymbol{x}; \theta_1, \theta_2, \ldots, \theta_m), \quad l \geq 1, \tilde{a}_j, b_j \sim O(1) \tag{36}$$

such that $\tilde{a}_j$ satisfies the relation (35) [11].

All of the analysis generalizes to the considered genetic switching case without any difficulty.

IV. INTRODUCTION OF THE GMAM

For the ease of readers, we here give a brief synopsis of the Geometric Minimum Action Method (gMAM), one can have a more detailed reading by reference [12].

The geometric minimum action method (gMAM) is a variant of the minimum action method (MAM)[13]. Both of them are going to minimize the action functional required to compute quasi-potential $V(x_1, x_2)$ in Freidlin-Wentzell theory and finding the minimizer.

For the presence of small random perturbations in dynamical systems, the behavior of the systems can't be described by the deterministic models all the time and the effect of the noise becomes ubiquitous. When event with very little likelihood occurs, large-deviations theory gives a rough estimate for the probability that the trajectory $X^{\varepsilon}(t), t \in$

$[0,T], T<\infty$, of the random dynamical system lies in a small neighborhood around a given path $\psi \in C(0,T)$, where $C(0,T)$ denotes the space of all continuous functions mapping from $[0,T]$ into $\mathbb{R}^n$. The theory asserts that, for σ and ε sufficiently small,

$$\mathbb{P}_x\{\sup_{0\le t\le T}|X^\varepsilon(t)-\psi(t)|\le\delta\}\approx\exp(-\varepsilon^{-1}S_T(\psi)), \tag{37}$$

where $\mathbb{P}_x$ denotes the probability conditioned on $X^\varepsilon(0)=x$ and assume $\psi(0)=x$. The action can be written as

$$S_T(\psi)=\begin{cases}\int_0^T L(\psi,\dot\psi)dt & \text{if } \psi\in C(0,T) \text{ is absolutely continuous and the integral converges,}\\ +\infty & \text{otherwise,}\end{cases} \tag{38}$$

where the Lagrangian $L(x,y)$ is given by

$$L(x,y)=\sup_{\theta\in\mathbb{R}^n}(\langle y,\theta\rangle-H(x,\theta)). \tag{39}$$

Here $\langle\cdot,\cdot\rangle$ denotes the Euclidean scalar product in $\mathbb{R}^n$ and $H(x,\theta)$ is the Hamiltonian.

One can obtain the quasi-potential of a system by minimize its action functional

$$V(x_1,x_2)=\inf_{T>0}\inf_{\psi\in\bar C_{x_1}^{x_2}(0,T)}S_T(\psi), \tag{40}$$

where $\bar C_{x_1}^{x_2}(0,T)$ denotes the space of all absolutely continuous functions $f:[0,T]\to\mathbb{R}^n$ such that $f(0)=x_1$ and $f(T)=x_2$. This is a double minimization problem. The key idea behind the gMAM is to reformulate the Freidlin-Wentzell action functional on the space of curves, thus eliminates the minimization problem to only the spacial dimension, instead of the double minimization on both spacial and temporal dimensions in MAM.

Now we skip the proof and go straight to the essence and algorithm of the gMAM. The key idea is to reformulate (40) geometrically in terms of curves $\gamma=\{\varphi(\alpha)\mid\alpha\in[0,1]\}$, where $\varphi:[0,1]\to\mathbb{R}^n$ is an arbitrary parametrization of the curve γ. The main result in this direction is that the quasi-potential (40) can be expressed as

$$V(x_1,x_2)=\inf_{\varphi\in\bar C_{x_1}^{x_2}(0,1)}\hat S(\varphi)\quad\text{with }\hat S(\varphi)=\sup_{\vartheta:[0,1]\to\mathbb{R}^n,H(\varphi,\vartheta)\equiv0}\int_0^1\langle\varphi',\vartheta\rangle d\alpha \tag{41}$$

$$=\int_0^1\langle\varphi',\hat\vartheta(\varphi,\varphi')\rangle d\alpha \tag{42}$$

$$=\int_0^1\frac{L(\varphi,\lambda\varphi')}{\lambda}d\alpha,\quad\lambda=\lambda(\varphi,\varphi'). \tag{43}$$

Here, the functions $\hat\vartheta(x,y)$ is implicitly defined for all $x\in D$ and $y\in\mathbb{R}^n\backslash\{0\}$ as the unique solution $\hat\vartheta,\lambda\in\mathbb{R}^n\times[0,\infty)$ of the system

$$H(x,\hat\vartheta)=0,\quad H_\theta(x,\hat\vartheta)=\lambda y,\quad\lambda\ge0. \tag{44}$$

As to our problem, Freidlin-Wentzell theory only provide the form of Hamiltonian. Though for some special cases, such as SDE

$$dX^\varepsilon(t)=b(X^\varepsilon(t))dt+\sqrt{\varepsilon}\sigma(X^\varepsilon(t))dW(t), \tag{45}$$

the form of Lagrangian can be solved explicitly by Legendre transform of Hamiltonian, there is no close form of Lagrangian generally. So the algorithm of gMAM can be expressed in two steps. The first is solve (44) to have $\hat\vartheta$ and λ, we call it innerloop. The second it that assuming $\hat\vartheta$ and λ are known, design a steepest-decent algorithm to solve Euler-Lagrange equation, we call it outerloop. Of course, if explicit form of Lagrangian is available, innerloop step can be ignored.

A. The Outer Loop

The Euler-Lagrange equation of variational problem (41) is given by([12]):

$$\begin{cases}-\lambda^2\varphi''+\lambda H_{\theta x}\varphi'-H_{\theta\theta}H_x-\lambda\lambda'\varphi'=0\\ \varphi(0)=x_1,\varphi(1)=x_2.\end{cases} \tag{46}$$

where$\lambda = \frac{\langle H_\theta, \varphi' \rangle}{|\varphi'|^2}$, and $H_x, H_\theta, H_{\theta\theta}$ are evaluated at $(\varphi', \hat{\vartheta}(\varphi, \varphi'))$.

In the steepest-descent algorithm, we use τ to represent time and α still the curve parameter. First we discretize $\varphi(\tau, \alpha)$ both in τ and α; i.e., we define $\varphi_i^k = \varphi(k\Delta\tau, i\Delta\alpha), k \in \mathbb{N}_0, i = 0, \ldots, N$, where $\Delta\tau$ is the time step and $\Delta\alpha = 1/N$ if we discretize the curve into $N + 1$ points. Then we discretize the initial condition $\varphi(0, \alpha)$ to obtain $\{\varphi_i^0\}_{i=0,\ldots,N}$ and, for $k \geq 0$, use the following two-step method to update these points:

(1) Given φ_i^k, compute ${\varphi'}_i^k = (\varphi_{i+1}^k - \varphi_{i-1}^k)/(2/N), \hat{\vartheta}_i^k = \hat{\vartheta}(\varphi_i^k, {\varphi'}_i^k)$, and $\lambda_i^k = \langle H_\theta(\varphi_i^k, \hat{\vartheta}_i^k), {\varphi'}_i^k \rangle / |{\varphi'}_i^k|^2$ for $i = 1, \ldots, N-1$, and set $\lambda_0^k = 3\lambda_1^k - 3\lambda_2^k + \lambda_3^k$ and $\lambda_N^k = 3\lambda_{N-1}^k - 3\lambda_{N-2}^k + \lambda_{N-3}^k$. Finally, compute ${\lambda'}_i^k = (\lambda_{i+1}^k - \lambda_{i-1}^k)/(2/N)$ for $i = 1, \ldots, N-1$.

(2) Let $\{\tilde{\varphi}_i\}_{i=0,\ldots,N}$ be the solution of the linear system

$$\begin{cases} \dfrac{\tilde{\varphi}_i - \varphi_i^k}{\Delta\tau} = (\lambda_i^k)^2 \dfrac{\tilde{\varphi}_{i+1} - 2\tilde{\varphi}_i + \tilde{\varphi}_{i-1}}{1/N^2} - \lambda_i^k H_{\theta x} {\varphi'}_i^k + H_{\theta\theta} H_x + \lambda_i^k {\lambda'}_i^k {\varphi'}_i^k, \quad i = 1, \ldots, N-1, \\ \tilde{\varphi}_0 = x_1, \\ \tilde{\varphi}_N = x_2, \end{cases} \tag{47}$$

where $H_{\theta x}, H_{\theta\theta}$, and H_x are evaluated at $(\varphi_i^k, \hat{\vartheta}_i^k)$.

(3) Interpolate a curve across $\{\tilde{\varphi}_i\}_{i=0,\ldots,N}$ and discretize this curve to find $\{\varphi_i^{k+1}\}_{i=0,\ldots,N}$ so that the prescribed constraint on the parametrization of φ is satisfied.

(4) Repeat until some stopping criterion is fulfilled.

B. Evaluating the Action

After the outer loop above, one can easily compute the value of the action by adding the following steps:

(5) Given $\{\varphi_i^k\}_{i=0,\ldots,N}$, compute ${\varphi'}_i^k, \hat{\vartheta}_i^k$, and λ_i^k as in Step 1 for every $i = 0, \ldots, N$.

(6) Return the action

$$\hat{S} = \frac{1}{N}\Big(\frac{3}{2}\langle {\varphi'}_i^k, \hat{\vartheta}_1^k \rangle + \sum_{i=2}^{N-2} \langle {\varphi'}_i^k, \hat{\vartheta}_i^k \rangle + \frac{3}{2}\langle {\varphi'}_{N-1}^k, \hat{\vartheta}_{N-1}^k \rangle\Big). \tag{48}$$

C. The Inner Loop (Computing $\hat{\vartheta}(\varphi, \varphi')$)

In order to compute $\hat{\vartheta}(\varphi, \varphi')$ from (44), we use function $h(\cdot)$ to denote the strictly convex and twice-differentiable function $H(\varphi, \cdot)$. The quadratically convergent routine is as follows. For $p \geq 0$:

(1)

$$\hat{\vartheta}^{p+1} := \hat{\vartheta}^p + h_{\theta\theta}^{-1}\big(\tilde{\lambda}(\hat{\vartheta}^p)\varphi' - h_\theta\big) \quad \text{with } \tilde{\lambda}(\hat{\theta}^p) := \left(\frac{\langle h_\theta, h_{\theta\theta}^{-1} h_\theta \rangle - 2h}{\langle \varphi', h_{\theta\theta}^{-1} \varphi' \rangle}\right)_+^{1/2}, \tag{49}$$

where $w_+^{1/2} = \sqrt{w}$ if $w \geq 0$ and $w_+^{1/2} = 0$ otherwise, and where h, h_θ, and $h_{\theta\theta}$ are evaluated at $\hat{\vartheta}^p$.

(2) Repeat until convergence.

It is shown in [12] that this algorithm guarantees local quadratic convergence. So when applying this algorithm, we need a proper initial guess of ϑ. However, we usually have little knowledge about ϑ. If one find in a particular problem, innerloop does not converge, we suggest the following improvement.

Equation (44) can be viewed as Lagrange multiplier method that solves constrained optimization problem:

$$\begin{cases} \max \langle \vartheta, \varphi' \rangle \\ s.t. \quad h(\vartheta) = 0 \end{cases} \tag{50}$$

So we can first apply a quadratic penalty method on this optimization problem. That is, we solve unconstrained problem

$$\min \frac{1}{2}\mu h^2(\vartheta) - \langle \vartheta, \varphi' \rangle \tag{51}$$

where μ is a penalty factor. We can simply use Newton method with a proper line search to solve this problem. We know from [14] that Newton method with exact line search guarantees global convergence. Although this step may be a little expansive in computing, we need only a few step to offer the original innerloop a proper initial value. It is also easy to show that this strategy guarantees global convergence.

V. STOCHASTIC SIMULATION

To check our theoretical predictions, we performed Monte Carlo (MC) simulations using the Gillespie algorithm [15] and implemented it in Microsoft Visual C++ Express 2010.

A. Transition Trajectory from MC Simulation

The value of each parameter chosen for simulation are: $k_0 = 1$, $r_0 = 50, a_0 = 0.4$, $a = 400$, $b = 40$, $\gamma_m = 10$, $\gamma_n = 1$, $k_1 = 0.0002$, and $\gamma_1 = 2$. With these parameters, the mean numbers of mRNA (m) , protein (n) and dimer (d) obtained from the deterministic equations are: $m_{off} = 0.04, m_{on} = 29.4, n_{off} = 1.6, n_{on} = 1175.1, d_{off} = 0.0003, d_{on} = 138.1$. There are two kinds of typical criterion for switch in MC simulation. One is to check whether the system has passed through the stable manifold of the saddle-node point, the other is to define two small boxes around each stable point and check whether the system has jumped from one box to the other. Because between two switches, most of the time is wasted in randomly walking around one of the two stable points and after passing the stable manifold of the saddle-node point, the system converges quickly to the corresponding stable point. Therefore, this two kinds of criterion mentioned above is almost the same. Here for convenience, in Fig. 2 we adopted the second kind of criterion and in Fig. 4 the first kind. The two boxes are defined as below: {off: $m_{off} \pm 2.5$, $n_{off} \pm 20.0$, $d_{off} \pm 3.5$} and {on: $m_{on} \pm 5.0$, $n_{on} \pm 70.0$, $d_{on} \pm 10.$} We use the following method to record each transition trajectory:

(1) Determine the system being in which box.

(2) Wait until the system goes out of this box, and note down the trajectory to a memory unit.

(3) If the system comes back to the same box, free the memory unit and goes to step (2); if the system goes into the other box, output the transition trajectory in the memory unit and free it.

(4) Repeat from step (2) until the stopping criterion is fulfilled.

B. Mean Switching Time (MST) from MC simulation

Here we use Mean First-Passage Time (MFPT) to approximately represent MST. Because the system is three dimensional, in each set of parameters we first set the system at the corresponding initial state, and run MC simulation until the system crosses the saddle node (thus crosses the stable manifold of saddle node). For each set of parameters, we run MC simulation 1000 times.

C. Local Property: Fluctuation around Stable States

As to the inconsistent portion between analytical and simulation results (the left part of the line with slow promoter transition rates in Fig. 5A), the reason is below. In Fig. 5, from right to left the high stable state is losing its stability. During this process, the standard deviation is increasing while the distance between high stable point and saddle point is decreasing. Because our analytical results were based on the local information around high stable state, and the MC simulation results were constricted by the stability of the high stable state. Therefore the inconsistency becomes apparent when the noise is high.

VI. ANALYSIS OF UPHILL PATH

We will do more analysis about uphill path for both diffusion process and chemical jump process. Recall that that the uphill path has the form

$$\dot{\boldsymbol{x}} = \nabla_{\boldsymbol{p}} H(\boldsymbol{x}, \nabla_{\boldsymbol{x}} S). \tag{52}$$

For the diffusion process

$$\dot{\boldsymbol{\varphi}} = \boldsymbol{b}(\boldsymbol{\varphi}) + \sqrt{\varepsilon}\boldsymbol{\sigma}(\boldsymbol{\varphi}) \cdot \dot{\boldsymbol{w}} \tag{53}$$

where $\boldsymbol{w}_j$ are independent temporal Gaussian white noise with properties $\mathbb{E}\dot{\boldsymbol{w}}_j(t) = 0$, $\mathbb{E}\dot{\boldsymbol{w}}_j(t)\dot{\boldsymbol{w}}_k(s) = \delta_{jk}\delta(t-s)$. The Hamiltonian is $H(\boldsymbol{\varphi}, \boldsymbol{p}) = \boldsymbol{b}(\boldsymbol{\varphi}) \cdot \boldsymbol{p} + \frac{1}{2}\boldsymbol{p}^T \cdot \boldsymbol{a} \cdot \boldsymbol{p}$ and the diffusion matrix $\boldsymbol{a}(\boldsymbol{\varphi}) = \boldsymbol{\sigma} \cdot \boldsymbol{\sigma}^T$. With this specific form we obtain the uphill path

$$\dot{\boldsymbol{x}} = \boldsymbol{b}(\boldsymbol{x}) + \boldsymbol{a} \cdot \nabla_{\boldsymbol{x}} S(\boldsymbol{x}). \tag{54}$$

If the drift $\boldsymbol{b}$ has the decomposition $\boldsymbol{b}(\boldsymbol{x}) = -\boldsymbol{a} \cdot \nabla_{\boldsymbol{x}} U(\boldsymbol{x}) + \boldsymbol{l}(\boldsymbol{x})$ such that $\boldsymbol{l}(\boldsymbol{x}) \cdot \nabla_{\boldsymbol{x}} U = 0$, then we have the result $S(\boldsymbol{x}) = 2U(\boldsymbol{x})$[16], which gives the rationale why $S(\boldsymbol{x})$ is called quasi-potential[16]. This orthogonal decomposition also tells that the uphill path is given by $\dot{\boldsymbol{x}} = \boldsymbol{a}(\boldsymbol{x}) \cdot \nabla U(\boldsymbol{x}) + \boldsymbol{l}(\boldsymbol{x})$.

For the general chemical jump processes, the similar picture still holds, but the argument on the orthogonal type decomposition of the drift is no longer valid if we recall that the uphill dynamics is $\dot{\boldsymbol{x}} = \nabla_{\boldsymbol{p}} H(\boldsymbol{x}, \nabla_{\boldsymbol{x}} S)$. In general, this form does not permit to specify some matrix $\boldsymbol{a}$ and make a meaningful decomposition because of full nonlinearity of H. However, it is instructive to do analysis under the condition that $\boldsymbol{p}$ is small. Recall that the Hamiltonian of a chemical jump process has the form

$$H(\boldsymbol{x}, \boldsymbol{p}) = \sum_{j=1}^{M} a_j\,(\boldsymbol{x})(e^{\boldsymbol{p}\cdot\boldsymbol{\nu}_j} - 1)\,, \tag{55}$$

thus the uphill path

$$\dot{\boldsymbol{x}} = \sum_{j=1}^{M} a_j(\boldsymbol{x})\boldsymbol{\nu}_j e^{\nabla_{\boldsymbol{x}} S\cdot\boldsymbol{\nu}_j} = \boldsymbol{b}(\boldsymbol{x}) + \sum_{j=1}^{M} a_j(\boldsymbol{x})\boldsymbol{\nu}_j\left(e^{\nabla_{\boldsymbol{x}} S\cdot\boldsymbol{\nu}_j} - 1\right) \tag{56}$$

where $\boldsymbol{b}(\boldsymbol{x}) = \sum_{j=1}^{M} a_j(\boldsymbol{x})\boldsymbol{\nu}_j$ is exactly the right hand side of the deterministic mean field dynamics. If $\boldsymbol{p}$ is small, we can make the approximation

$$e^{\nabla_{\boldsymbol{x}} S\cdot\boldsymbol{\nu}_j} \approx 1 + \nabla_{\boldsymbol{x}} S \cdot \boldsymbol{\nu}_j, \tag{57}$$

then (56) leads to

$$\dot{\boldsymbol{x}} = \boldsymbol{b}(\boldsymbol{x}) + \sum_{j=1}^{M} a_j(\boldsymbol{x})\boldsymbol{\nu}_j \otimes \boldsymbol{\nu}_j \cdot \nabla_{\boldsymbol{x}} S. \tag{58}$$

This is exactly the uphill path for chemical Langevin dynamics

$$\dot{\boldsymbol{\varphi}} = \sum_{j=1}^{M} a_j(\boldsymbol{\varphi})\boldsymbol{\nu}_j + \sum_{j=1}^{M} \sqrt{a_j(\boldsymbol{\varphi})}\boldsymbol{\nu}_j \cdot \dot{\boldsymbol{w}}_j, \tag{59}$$

This analysis tells us that near the critical points, that is, the optimal uphill path of a chemical jumping process can be well approximated by the uphill path of a chemical Langevin dynamics. The situation with DNA fast switching is discussed in the main text.

VII. APPLICATION IN TRANSCRIPTIONAL CASCADES

The model we used was based on the previous work of Sara Hooshangi and Ron Weiss, etc[17]. In their work, they synthesized transcriptional cascades comprising one, two, and three repression stages and analyzed the sensitivity and noise propagation as a function of network complexity. The original gene network they used is shown in Fig. S1.

FIG. S1: The network design of three synthetic transcriptional cascades[17]. In all circuits, TetR is expressed constitutively from P_{lacIq} promoter. aTc, which freely diffuses into the cell, binds TetR and prevents the repression of $P_{Ltet-O1}$. (A) In the one-stage cascade (circuit 1), *eyfp* expression is under the control of TetR repressor. (B) Circuit 2 has an additional repression stage where the expression of *eyfp* is controlled by LacI protein, which can be repressed by the TeR repressor. (C) In circuit 3, *eyfp* expression is controlled by the CI repressor. *cI* expression is controlled by LacI protein, which is under the control of TetR.

A. Step 1: Deterministic Model Described by the ODEs

To further illustrate the powerful visual effect of quasi-potential energy landscape and the abundant quantitative information it contains, here we simplify the network by modeling each stage as a Gillespies birth-death process and reaction rate of each stage is governed by a Hill function. The simplified model is

$$x \to y_1 \dashv y_2 \dashv y_3$$

Here, x denotes the concentration of original inducing signal atc, and $y_1,\ y_2,\ y_3$ correspond to the concentration of GFP in each cascade, i.e. the expression level of protein *LacI, CI, Eyfp,* respectively. This model involves 9 reactions and we state the model in Table II as below.

TABLE II: The list of reactions in the transcriptional cascades model

Reaction	Propensity Function	Stoichiometric Vector
$\emptyset \longrightarrow y_1$	a_0	$(1,0,0)$
$\emptyset \longrightarrow y_2$	a_0	$(0,1,0)$
$\emptyset \longrightarrow y_3$	a_0	$(0,0,1)$
$x \longrightarrow y_1$	$f_1(x)$	$(1,0,0)$
$y_1 \longrightarrow y_2$	$f_2(y_1)$	$(0,1,0)$
$y_2 \longrightarrow y_3$	$f_3(y_2)$	$(0,0,1)$
$y_1 \longrightarrow \emptyset$	$\gamma_1 y_1$	$(-1,0,0)$
$y_2 \longrightarrow \emptyset$	$\gamma_2 y_2$	$(0,-1,0)$
$y_3 \longrightarrow \emptyset$	$\gamma_3 y_3$	$(0,0,-1)$

Here $\gamma_i = 1$ $(i = 1,2,3)$, $f_i(u)$ are Hill functions with form

$$f_i(u) = \frac{(a_i - a_0)\left(\dfrac{u}{K_i}\right)^n}{1+\left(\dfrac{u}{K_i}\right)^n}, \quad i = 1,2,3.$$

x is a parameter used to denote the concentration of aTc, a_0 the basal production rate of each protein, a_i the maximum production rate of each protein, y_i the output concentration in circuit i, K_i the microscopic dissociation constant, $i = 1,2,3$, and n the Hill coefficient. The values of parameters are $a_0 = 1,\ n = 2,\ a_i = 101,\ K_i = 10,\ i = 1,2,3$. The corresponding deterministic equations are given by

$$\begin{aligned}
\frac{dy_1}{dt} &= a_0 + \frac{(a_1 - a_0)\left(\dfrac{x}{K_1}\right)^n}{1+\left(\dfrac{x}{K_1}\right)^n} - y_1,\\
\frac{dy_2}{dt} &= a_0 + \frac{a_2 - a_0}{1+\left(\dfrac{y_1}{K_2}\right)^n} - y_2,\\
\frac{dy_3}{dt} &= a_0 + \frac{a_3 - a_0}{1+\left(\dfrac{y_2}{K_3}\right)^n} - y_3.
\end{aligned} \tag{60}$$

B. Step 2: Hamiltonian Derived from the Large Deviation Theory

The stochastic model is the Gillespie type birth-death process without DNA switching. Recall routines stated in section II and equation (17), the Hamiltonian of this system is given by

$$H(\boldsymbol{y},\boldsymbol{p}) = \sum_{i=1}^{3} a_0(e^{p_i}-1) + f_i(y_{i-1})(e^{p_i}-1) + (e^{-p_i}-1), \tag{61}$$

where $\boldsymbol{y} = (y_1, y_2, y_3), \boldsymbol{p} = (p_1, p_2, p_3)$. Here we denote $y_0 \equiv x$.

C. Step 3: Constructing the Quasi-potential Energy Landscape

Here we skip the process of computation and directly give the final results. Detailed information of gMAM may be found in section IV. In Fig. 6, we show the The probability distribution of the final output as a function of inducing signal concentration with different depths of cascades. Here we use the information from Fig. 6 to directly compute the coefficient of variation (CV) as a function of mean output (see Fig. S2). We also show the MC simulation results of our simplified model described by Eq. (60). From Fig. S2 we can see that as the total layers of cascades increase, the corresponding fluctuation of output (described by CV) increases, especially when the level of output is in its intermediate state.

The Hill coefficient for each response curve in Fig. 6 is calculated as follows. In each subfigure of Fig. 6, we find out the maximum output value induced by each input signal, then we have three functions $y_1(x),\ y_2(x),\ y_3(x)$. Actually, they are consistent with the results by setting each equation to zero in Eq. (60). Then for $i = 1, 2, 3$, we have

$$\begin{aligned} y_i(x_1^{(i)}) &= 0.1(a_1 - a_0) + a_0, \\ y_i(x_2^{(i)}) &= 0.9(a_1 - a_0) + a_0. \end{aligned}$$

Thus the Hill coefficient n for each cascade is

$$n^{(1)} = \frac{\ln 81}{\ln\left(\dfrac{x_2^{(1)}}{x_1^{(1)}}\right)}, \quad n^{(2)} = \frac{\ln 81}{\ln\left(\dfrac{x_1^{(2)}}{x_2^{(2)}}\right)}, \quad n^{(3)} = \frac{\ln 81}{\ln\left(\dfrac{x_2^{(3)}}{x_1^{(3)}}\right)}.$$

FIG. S2: Coefficient of variation as a function of mean output is used to show the fluctuation in the transition region. Analytical results obtained directly from Fig. 6 is shown in solid line, while MC simulation is shown in discrete markers. Circuit 1 corresponds to red solid line and red square, circuit 2 magenta solid line and magenta circle, circuit 3 blue solid line and blue diamond.

---

1. G. Papanicolaou, *Introduction to the asymptotic analysis of stochastic equations*, Lectures in Mathematics, Vol. 16, American Mathematical Society, Rode Island, 1977.
2. M. Assaf, E. Roberts, E. Luthey-Schulten, Determining the stability of genetic switches: Explicitly accounting for mRNA noise, Phys. Rev. Lett. 106 (2011), 248102.
3. E. Roberts, et al. Noise Contributions in an Inducible Genetic Switch: A Whole-Cell Simulation Study, PLoS Comput. Biol. 7 (2011), e1002010.
4. J. T. Gerstle, M. G. Fried, Measurement of binding kinetics using the gel electrophoresis mobility shift assay. Electrophoresis, 8 (1993), 725-731.
5. Y. Taniguchi, et al. Quantifying E. coli proteome and transcriptome with single-molecule sensitivity in single cells. Science. 5991 (2010), 533-538.
6. D. Kolodrubetz, R. Schleif., Identification of araC protein and two-dimensional gels, its in vivo instability and normal level. J. Mol. Biol. 1(1981), 133-139.
7. J. Yu, et al. Probing gene expression in live cells, one protein molecule at a time. Science, 5767 (2006), 1600-1603.
8. H. C. Tsui, G. Feng, M. E. Winkler, Negative regulation of mutS and mutH repair gene expression by the Hfq and RpoS global regulators of Escherichia coli K-12. J. Bacteriol. 23 (1997), 7476-7487.

[9] Liptser R.S. (1996) Large Deviation for Two Scaled Diffusion Probab. Theory Relat. Fields 106, 71-104
[10] T. Li, F. Lin and Y. Liu (2013) Large Deviation for a class of jump processes, preprint.
[11] T.G. Kurtz, The relationship between stochastic and deterministic models for chemical reactions. J. Chem. Phys. 57 (1972), 2976.
[12] M. Heymann and E. Vanden-Eijnden, The geometric minimum action method: a least action principle on the space of curves, Comm. Pure Appl. Math. 61 (2008), 1052-1117.
[13] W. E, W. Ren and E. Vanden-Eijnden, Minimum action method for the study of rare events, Comm. Pure Appl. Math. 57 (2004), 637-656.
[14] Jorge Nocedal and Stephen J. Wright, Numerical Optimization, Springer, 2nd Edition, 2006
[15] D. T. Gillespie, exact stochastic simulation of coupled chemical reactions, J. Phys. Chem. 81 (1977), 2340.
[16] M.I. Freidlin and A.D. Wentzell, *Random perturbations of dynamical systems*, Springer-Verlag, New York, 2nd Edition, 1998.
[17] Hooshangi S, Thiberge S, R Weiss (2005) Ultrasensitivity and noise propagation in a synthetic transcriptional cascade. *Proc Natl Acad Sci USA* 102(10):3581-3586.
